# Mesenchymal stem cells and their derivatives as potential longevity-promoting tools

**DOI:** 10.1007/s10522-025-10240-z

**Published:** 2025-04-21

**Authors:** Ekaterina Rudnitsky, Alex Braiman, Marina Wolfson, Khachik K. Muradian, Vera Gorbunova, Gadi Turgeman, Vadim E. Fraifeld

**Affiliations:** 1 The Shraga Segal Department of Microbiology, Immunology and Genetics, Faculty of Health Sciences, Ben-Gurion University of the Negev, P.O.Box 653, 8410501 Beer-Sheva, Israel; 2 Department of Biology of Aging and Experimental Life Span Extension, State Institute of Gerontology of National Academy of Medical Sciences of Ukraine, Kiev, 4114 Ukraine; 3 Department of Biology, University of Rochester, Rochester, NY 14627 USA; 4 Department of Medicine, University of Rochester Medical Center, Rochester, NY 14627 USA; 5 Department of Molecular Biology, Faculty of Natural Sciences and Medical School, Ariel University, 40700 Ariel, Israel

**Keywords:** MSCs, Extracellular vesicles, Stem cells, Aging signatures, Cell therapy, Longevity

## Abstract

Mesenchymal stem cells (MSCs) and blood plasma/MSC-derived extracellular vesicles (EVs) offer promising tools to promote longevity and treat age-related diseases. MSCs have low immunogenicity and tumorigenicity, and their efficacy is relatively independent of the donor age in humans (but not in rodents). Systemic administration of MSCs and stem cell/blood-derived EVs modified the omic profiles of various organs of aged rodents towards the young ones. The application of EVs appears to be even more beneficial than MSCs. Remarkably, over 70% of microRNAs, which are over-presented in ESC-derived EVs, were found to target longevity-associated genes. Along with MSCs, other types of stem cells were reported to display health- and lifespan-extending effects. Pluripotent Muse cells, a specific subpopulation of MSCs, which possess a number of unique features, could be particularly relevant for promoting healthspan. The rejuvenation potential of MSCs, EVs, and Muse cells warrants further investigation in both animal models and clinical trials, using aging clocks for biological age determination as one of the endpoints.

## Introduction

Mesenchymal stem cells (MSCs) represent a distinct population of mesenchymal stromal cells, which (i) are able to adhere to plastic surfaces, (ii) express specific cell surface markers (CD73, CD90, and CD105, but not CD14, CD34, CD45, and HLA-DR), (iii) and are able to differentiate into osteogenic, chondrogenic, or adipogenic cell lineages in vitro (Kulus et al. 2021; Galderisi et al. 2022). It should be noted that MSC isolation yields heterogeneous, non-clonal cultures of stromal cells, including stem cells with diverse multipotent potential, committed progenitors, and differentiated cells (Galderisi et al. 2022). MSCs are found in virtually all organs of the adult organism, examined thus far (da Silva Meirelles et al. 2006). A rapidly growing body of evidence indicates the beneficial effects of systemic administration of MSCs or MSC-derived extracellular vesicles (EVs) in various pathological conditions, including age-related diseases (ARDs) (Guy and Offen 2020; Zhuang et al. 2021; Smolinská et al. 2023; Emmrich et al. 2024; Rudnitsky et al. 2024; Tombak et al. 2025). For example, the systemic administration of bone marrow-derived MSCs or MSC-derived EVs from young rodents increased hippocampal neurogenesis and improved cognitive function in aged animals (Gobshtis et al. 2017, 2021; Yu et al. 2018; Herman et al. 2021; Tfilin et al. 2023).

Longevity is the most general and integrative parameter for evaluating the therapeutic effects of any interventions (Lyu et al. 2024). Another integrative parameter directly related to life expectancy is biological age. Recently, its determination has become possible, using various biological aging clocks (Moqri et al. 2025; Muradian and Fraifeld 2024a). However, to date, a comprehensive analysis of the impact of MSCs/MSC-derived EVs on longevity, biological age, and aging phenotypes has not been conducted. With this in mind, in this review, we primarily focus on the effects of MSC or EV administration on the lifespan of wild-type or progeroid animals. Other types of stem cells and EV sources were also considered. Along with the health- and lifespan-extending effects, we discuss their putative mechanisms as well as the impact on biological age and aging omic signatures.

## The effects of stem cell/MSC/EV administration on lifespan in rodents

The effects of systemic administration of stem cells/MSCs/EVs on lifespan in rodents are summarized in Table 1. These effects were investigated in both naturally aging rodents and progeroid mice. The MSCs or EVs were delivered systemically (i.p., i.v., or into the left ventricular cavity of the heart), and the frequency of their administration varied from a single injection to weekly injections until natural death. The transplantation of MSCs or infusion of EVs led to a consistent extension of both median (or mean) and maximum lifespan in rodents. This was observed in both naturally aging and progeroid animals. Moreover, in the vast majority of studies, the lifespan-extending effects were accompanied by attenuation of aging symptoms, including preservation of physical activity, cognitive function, and metabolism (a decrease in insulin resistance and maintenance of bone mineral density) (Table 1).

**Table 1:** The effects of systemic administration of MSCs or blood-extracted EVs on lifespan in rodents

Origin of stem cells or EVs	Regimen of treatment	Animals (sex/#/age at the beginning of treatment)	Aging model	Effects	References
Bone marrow-derived MSCs from 1–2-month-old male C57BL/6 J mice	10^6^ MSCs i.v. per mouse, single injection	Balb/C mice ♀ n = 7 per group 18–24 months	Natural aging	Extension of median (by 15%) and maximum (by 6%) lifespan Preservation of bone mineral density	Shen et al. (2011)
Human amniotic membrane-derived MSCs (data on donors is not indicated)	10^6^ MSCs i.v. per rat, multiple injections once a month till natural death	F344 rats ♀ n = 20–30 per group 10 months	Natural aging	Extension of median (by 32%) and maximum (by 33%) lifespan Maintenance of physical activity and cognitive function	Kim et al. (2015)
Human adipose tissue-derived MSCs from one healthy 53-year-old female donor	10^6^ MSCs i.v. per rat, multiple injections once a month till natural death	F344 rats ♀ n = 20–30 per group 10 months	Natural aging	Extension of median (by 32%) and maximum (by 48%) lifespan Maintenance of physical activity and cognitive function	Kim et al. (2015)
Human bone marrow-derived MSCs one healthy 56-year-old female donor	5 × 10^6^ MSCs i.v. per rat, multiple injections every two weeks till natural death	Sprague Dawley rat ♀ n = 1 6 months	Natural aging	Extension of lifespan (by 38%) Maintenance of physical activity Absence of mammary tumors	Mansilla et al. (2016)
Human adipose tissue-derived MSCs (data on donors is not indicated)	Lysate from 10^5^ MSCs i.p. per kg of body weight, multiple injections three times per week till natural death	Sprague Dawley rats ♂ and ♀ n = 22–24 per group 12 months	Natural aging	Slight shortening average lifespan (by 8%) Decrease in body weight, bone and fat mass	Hsu et al. (2018)
Muscle-derived stem/progenitor cells from 14–21-day-old male f1 C57BL/6:FVB/n or C57BL/10 J mice	2–4 × 10^5^ MSCs i.p. per g body weight, single injection	*Ercc1*^−/−^ mice ♀ n = 8 per group 17 days	Progeria (*Ercc1*^−/−^ mice)	Extension of median (by 314%) and maximum (by 236%) lifespan (*Ercc1*^−/−^ mice)	Lavasani et al. (2012)
Muscle-derived stem/progenitor cells from 14–21-day-old male f1 C57BL/6:FVB/n or C57BL/10 J mice	2–4 × 10^5^ MSCs i.p. per g body weight, two injections during 6 weeks (*Ercc1* ^−/△^ mice)	*Ercc1*^−/△^ mice ♀ n = 8 per group 6–7 weeks	Progeria (*Ercc1*^−/△^ mice)	Delay of the onset of aging symptoms (dystonia, trembling, kyphosis, ataxia, muscle wasting, loss of vision, urinary incontinence and decreased spontaneous activity) Survival data are not available	Lavasani et al. (2012)
Amniotic membrane-derived MSCs from C57BL/6 J mice in the middle and late phases of normal pregnancy	10^7^ MSCs i.p. per mouse, four injections once a week for 4 weeks	*Bmi-1*^*−/−*^ mice n = 6 per group 2 days	Progeria (*Bmi-1*^*−/−*^ mice)	Extension of median (by 236%) and maximum (by 173%) lifespan Increase in body weight and overall size of the body, thymus, spleen and kidney Partial preservation of thymic function Amelioration of premature osteoporosis	Xie et al. (2015)
Bone marrow-derived MSCs exposed to oxidative stress (20% O_2_ for 48 h) from 3–20-week-old C57/Bl6J mice	10^6^ MSCs i.p. per mouse, single injection	*Ercc1*^−/−^ mice n ≥ 4 per group 10 days	Progeria (*Ercc1*^−/−^ mice)	Extension of median (by 286%) lifespan	Dorronsoro et al. (2021)
EVs from blood plasma of 4–12-month-old female C57BL/6 J mice	100 μl of EVs from 200 μl blood plasma i.p., multiple injections once per week till natural death	C57BL/6 J mice ♀ n = 11–12 per group 26 months	Natural aging	Extension of median (by 10%) and maximum (by 16%) lifespan Maintenance of physical activity	Yoshida et al. (2019)
EVs from blood plasma of 2-month-old male C57BL/6 J mice	360 μg EV protein i.v. per mouse, multiple injections once a week till natural death	C57BL/6 J mice ♂ n = 5 per group 20 months	Natural aging	Extension of median (by 12%) and maximum (by 21%) lifespan Alleviation of frailty Maintenance of endurance, physical activity, and cognitive function Preservation of fertility and litter size	Chen et al. (2024)
EVs obtained from cardiosphere-derived cells from neonatal (2-day-old) F344 rats	Injections into the left ventricular cavity: the first injection with an initial dose of 1.3 × 10^8^ EVs per g of body weight was followed by four injections with dose of 2.6 × 10^7^ EVs per g of body weight once a month for 4 months	F344 rats ♂ and ♀ n = 13–14 per group 22 months	Natural aging	Extension of median lifespan (by ~ 60%) Decrease in insulin resistance Attenuation of tissue fibrosis in heart, lungs, skeletal muscle and kidney	Grigorian Shamagian et al. (2023)

In general, these effects were not significantly influenced by the source of MSCs (bone marrow, adipose tissue, amniotic membranes, etc.). However, the comparative analysis conducted by Kim et al. (2015) revealed that adipose tissue-derived MSCs were more efficient in extending the lifespan, whereas MSCs from amniotic membranes were better at maintaining physical activity and cognitive function (Kim et al. 2015). This could be attributed to the observation that MSCs from different tissues exhibit slightly different properties. In particular, Heo et al. (2016) demonstrated that, compared to placenta- or umbilical cord-derived MSCs, the bone marrow- and adipose tissue-derived MSCs possess higher capacity for self-renewal and the potential to differentiate into other mesodermal cell lineages (adipocytes, osteoblasts, and chondroblasts), as well as more pronounced anti-inflammatory activity.

The lifespan-extending effect was also observed for EVs extracted from cardiospheres or blood plasma (Table 1), and this effect was comparable to that of MSC transplantation. It is worth noting that defining the cell source of EVs isolated from blood plasma is difficult. However, based on the study of adipose tissue-specific *Nampt* knockout mice, it was suggested that adipose tissue is a valuable contributor (Yoshida et al. 2019). The important point is that extracellular Nampt, which promotes NAD^+^ generation, is contained exclusively in EVs, originating presumably from adipose tissue (Yoshida et al. 2019). It appears that the lifespan-extending effect of blood plasma-derived EVs is primarily associated with modulation of NAD^+^ levels. Indeed, the age-related decline of NAD^+^ was ubiquitously observed, and its prevention was shown to extend the lifespan of both invertebrate (yeast, worms, flies) and vertebrate (rodents) organisms (Yaku et al. 2018).

Lifespan-extending effects of MSCs as well as EVs do not seem to be sex-, strain-, or even species-specific, but could be dependent on donor age (Table 1). Indeed, stem cells from young mice extended median lifespan in both naturally aging and progeroid mice. In contrast, MSCs from old or progeroid mice did not exert any significant effect on lifespan (Shen et al. 2011; Lavasani et al. 2012; Dorronsoro et al. 2021). This may be attributed to age-related alterations of MSC function in rodents (Kasper et al. 2009). Notably, the lifespan-extending effect was also observed in the case of MSC transplantation from middle-aged human donors to rats (Kim et al. 2015; Mansilla et al. 2016). No significant difference in ‘cellular fitness’ in vitro between bone marrow-derived MSCs from very young (infants and children < 6 years) and middle-aged (38–58 years) human donors was observed, except for a slightly lower rate of cell division in older vs. young donors (Lund et al. 2010). Similar results were reported by Liu et al. (2014) for MSCs from human donors above 60 years of age. Transplantation of these MSCs exhibited cardioprotective effects in the myocardial infarction rat model.

Another important issue is whether the efficacy of stem cell or EV therapy depends on the age of recipients. Of note, in all studies carried out thus far, the recipient animals were of relatively advanced ages. In mice, the treatment was started in animals of 18 months of age or older. In rats, the treatment was started no earlier than 12 months of age when animals are considered ‘middle-aged’ (Campos-Beltrán and Marshall 2021), and was conducted for 4 months or until natural death (Table 1). It would be attractive to speculate that the age of a recipient is not a barrier for beneficial effects of stem cell/MSC/EV therapy.

To summarize, systemic stem cell/MSC/EV administration exerted a clear lifespan-extending effect, and this effect was observed even if the treatment was started late in life. It seems that the tissue source of MSCs had only a slight impact on lifespan. Donor age was of critical value in rodents but not in humans. Not surprisingly, the longevity-promoting effect of MSC transplantation was much more pronounced in progeroid mice than in wild-type animals.

## Effects of stem cells/MSCs/EVs on aging signatures

Thus far, only a few studies have been undertaken to evaluate the effects of systemic administration of stem cells/MSCs/EVs on biological age or age-related omic profiles. To the best of our knowledge, there are only two papers in which aging epigenetic clocks were used to evaluate such effects (Sanz-Ros et al. 2022; Horvath et al. 2024). In both studies, EVs derived from either MSCs or blood plasma were used, and impressive results were obtained. Sanz-Ros et al. (2022) found that proteins extracted from MSC-derived exosomes of young mice decreased epigenetic age, prevented frailty, and improved healthspan in old mice. Horvath et al. (2024) transplanted the exosome fraction of swine blood plasma to old rats, resulting in a significant reversal of biological age and functional improvement of various organs.

**Fig. 1:**
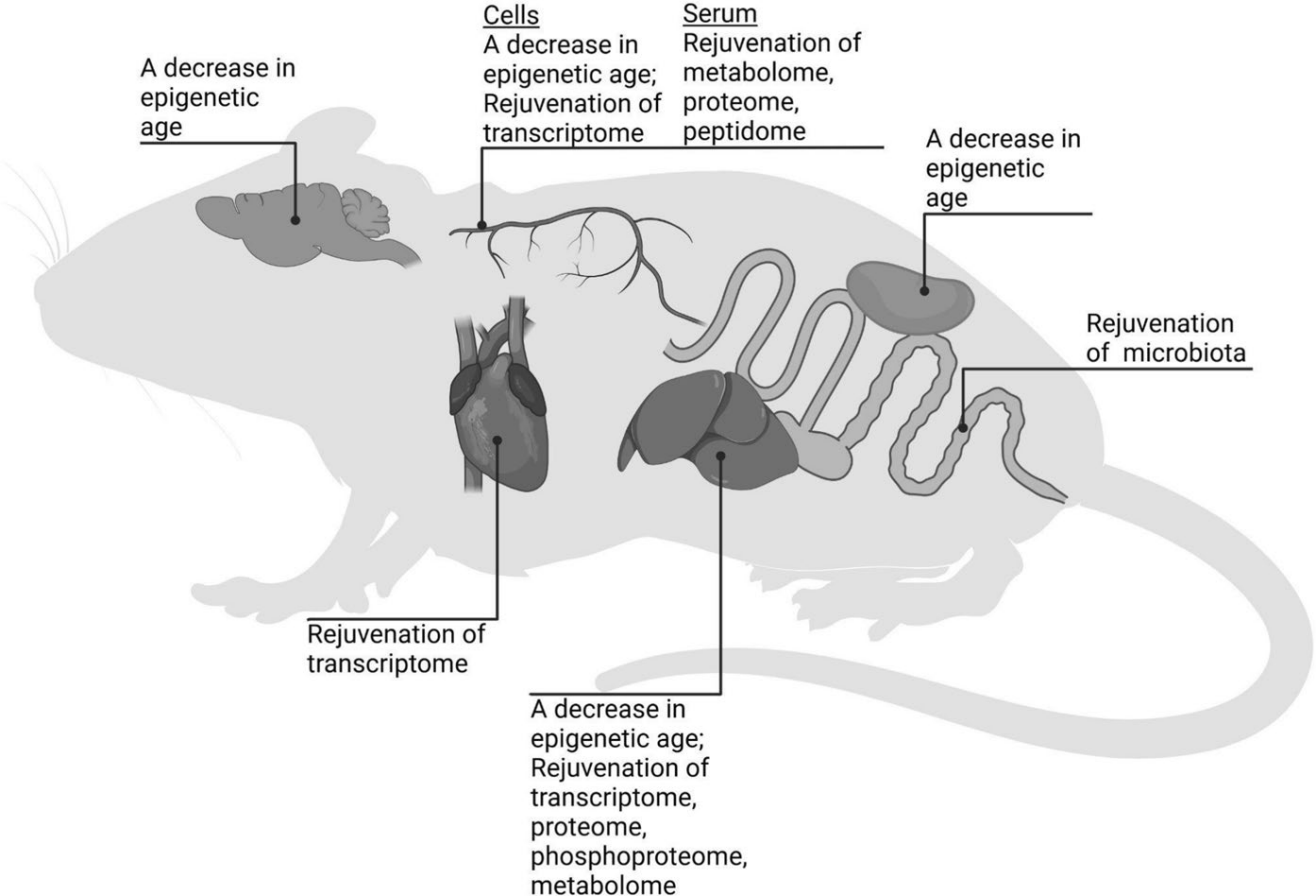
The effects of stem cell/MSC/EV systemic administration on aging signatures in various organs of rodents

In several studies, various age-related omic profiles (transcriptomic, metabolomic, proteomic, peptidomic, phosphoproteomic profiles, as well as gut microbiota) were evaluated after systemic stem cell/MSC/EV administration (Table 2). As a result, the omic profiles of various organs of aged rodents were modified, so that the profiles were comparable to those of younger animals. Indeed, the rejuvenative effects in liver, heart, brain, kidney, gut, and blood were observed (Fig. 1). In monkeys, transplantation of human ESC-derived MSC-like cells extended the reproductive lifespan (Yan et al. 2024). The rejuvenative effects of stem cells/MSCs/EVs do not seem to be species-specific (Table 2), similarly to the lifespan-extending experiments.

**Table 2:** The effects of systemic administration of MSCs or EVs on age-related changes in omics profiles

Stem cell or EV origin	Regimen of treatment	Animals (#/sex/age at the beginning of treatment)	Effects	References
Cardiosphere-derived cells from neonatal (2-day-old) Sprague Dawley rats	10^6^ cells per rat, into the left ventricular cavity or intramyocardially, single injection Follow-up period: 1 month	F344 rats n = 5–7 per group 21.8 ± 1.6 months	Transcriptomic profile of whole-heart extracts of cell-treated aged rats recapitulated transcriptomic profile of young rats	Grigorian Shamagian et al. (2017)
Human placenta-derived MSCs (data on donors is not indicated)	5 × 10^5^ MSCs i.v. per rat, three injections at 10-day intervals or 4-week intervals Follow-up period from the first injection: 2 months	Sprague Dawley rats ♀ n = 24 per group 52–54 weeks	Amelioration of aging-associated phenotype of metabolome of serum and liver	Kim and Lee (2022)
Adipose tissue-derived MSCs from 3–6-month-old C57BL/6 J mice	20 µg of EV protein i.v. per mouse, two injections, once a week Follow-up period from the first injection: 4 months	C57BL/6 J mice ♂ and ♀ n = 6 per group 20 months	Epigenetic age of EV-treated aged mice was lower in liver and kidney and did not differ in muscle and spleen compared to control aged mice Plasma metabolome of EV-treated aged mice recapitulated plasma metabolome of young mice	Sanz-Ros et al. (2022)
Human umbilical cord-derived MSCs (data on donors is not indicated)	10^9^ EVs i.v. per mouse, eight injections once a week Follow-up period from the first injection: 4 months	C57BL/6 J mice ♂ n = 6 per group 18 months	Amelioration of aging-associated phenotype of metabolome and phosphoproteome in liver	Ling et al. (2023)
Human bone marrow-derived MSCs from healthy young adult donors	10^6^ MSCs i.v. per mouse, single injection Follow-up period: 5 days	C57BL/6 J mice ♀ 24 months	Serum proteomic and peptidomic profiles of MSC-treated aged mice were less proinflammatory than serum proteomic and peptidomic profiles of untreated mice	Niu et al. (2023)
Human umbilical cord-derived MSCs (data on donors is not indicated)	5 × 10^6^ MSCs i.v. per rat, four injections once a week Follow-up period from the first injection: 5 weeks	Sprague Dawley rats ♂ n = 5 per group 24 months	Gut microbiota profile of MSC-treated aged rats recapitulated gut microbiota profile of young rats	Wang et al. (2023)
Human exfoliated deciduous teeth-derived stem cells from healthy 5–11-year-old donors	5 × 10^5^ i.v. cells per mouse, 12 injections once in two weeks Follow-up period from the first injection: 6 months	C57BL/6 J mice ♀ n = 15 per group 24 months	Transcriptomic and proteomic profiles of liver in stem cell-treated aged mice recapitulated transcriptomic and proteomic profiles of young mice	Xing et al. (2023)
Mouse ESC line D3	100 µg EVs i.p. per mouse, multiple injections every alternate day for 8 weeks Follow-up period from the first injection: 2 months	C57BL/6 J mice ♂ n = 3 per group 14 months	Transcriptomic profile of peripheral blood monocytes of EV-treated aged mice recapitulated transcriptomic profile of young mice	Yu et al. (2023)
The exosome fraction (E5) of platelet-free blood plasma from 6-month-old pigs	1.43 g of solid precipitate of exosomes per 500 g body weight; two series of four i.v. injections every alternate day for 8 days: first started at day 1, second started at day 95 Follow-up period from the first injection: 5 months	Sprague Dawley rats ♂ n = 18 per group 24 months	Progressive improvement in organ functions: a decrease in blood bilirubin, glucose, creatinine, cholesterol, pro-inflammatory cytokines to the levels of young animals Improvement of cognitive functions Reversal of biological age according to six different epigenetic clocks	Horvath et al. (2024)
Human ESC-derived MSC-like cells	5 × 10^6^ cells per ovary, two injections once a month Follow-up period from the first injection: 26 months	Cynomolgus monkeys (*Macaca fascicularis*) ♀ n = 10 (3 control, 7 treated) 18–23 years	Mitigation of inflammation, fibrosis, oxidative damage, and apoptosis in perimenopausal ovaries Elevated secretion of sex hormones in perimenopausal ovaries	Yan et al. (2024)

All in all, the “younger” state of various organs in stem cell/MSC/EV-treated aged animals may, to some extent, explain the lifespan-extending effects of such a therapy.

## Putative longevity-promoting mechanisms of stem cell/MSC/EV treatment

The accumulated body of evidence indicates that the transplanted MSCs could exert their beneficial effects on longevity and health by their secretome which includes soluble molecules and EVs (Siraj et al. 2023). In particular, the secretome of non-senescent MSCs revealed anti-inflammatory and anti-apoptotic properties. Whatever the case, the transplanted MSCs display their effects in a paracrine manner rather than by differentiating into other cell types (Boregowda and Phinney 2013; Francisco et al. 2019; Govindasamy et al. 2021). The *comparable* effects of MSCs and MSC-derived EVs strongly support this concept. Relevant issues are discussed in detail in the recent review by Li et al. (2023). Yet, EVs have some advantages over MSCs: EVs are much less immunogenic and fully non-tumorigenic (Tolar et al. 2007; Ali et al. 2024; Hye et al. 2024).

A prominent exception to the predominant paracrine mode of action of MSCs is their subpopulation denoted as Muse cells (multilineage-differentiating stress-enduring stem cells) (Kuroda et al. 2010). In contrast to other adult somatic stem cells, transplanted Muse cells successfully differentiate into various cell types (Kushida et al. 2018) and selectively home to damaged sites after systemic administration (Minatoguchi et al. 2024). Their beneficial therapeutic effects were shown in several models of ARDs (Alanazi et al. 2023; Velasco et al. 2023; Minatoguchi et al. 2024).

**Fig. 2:**
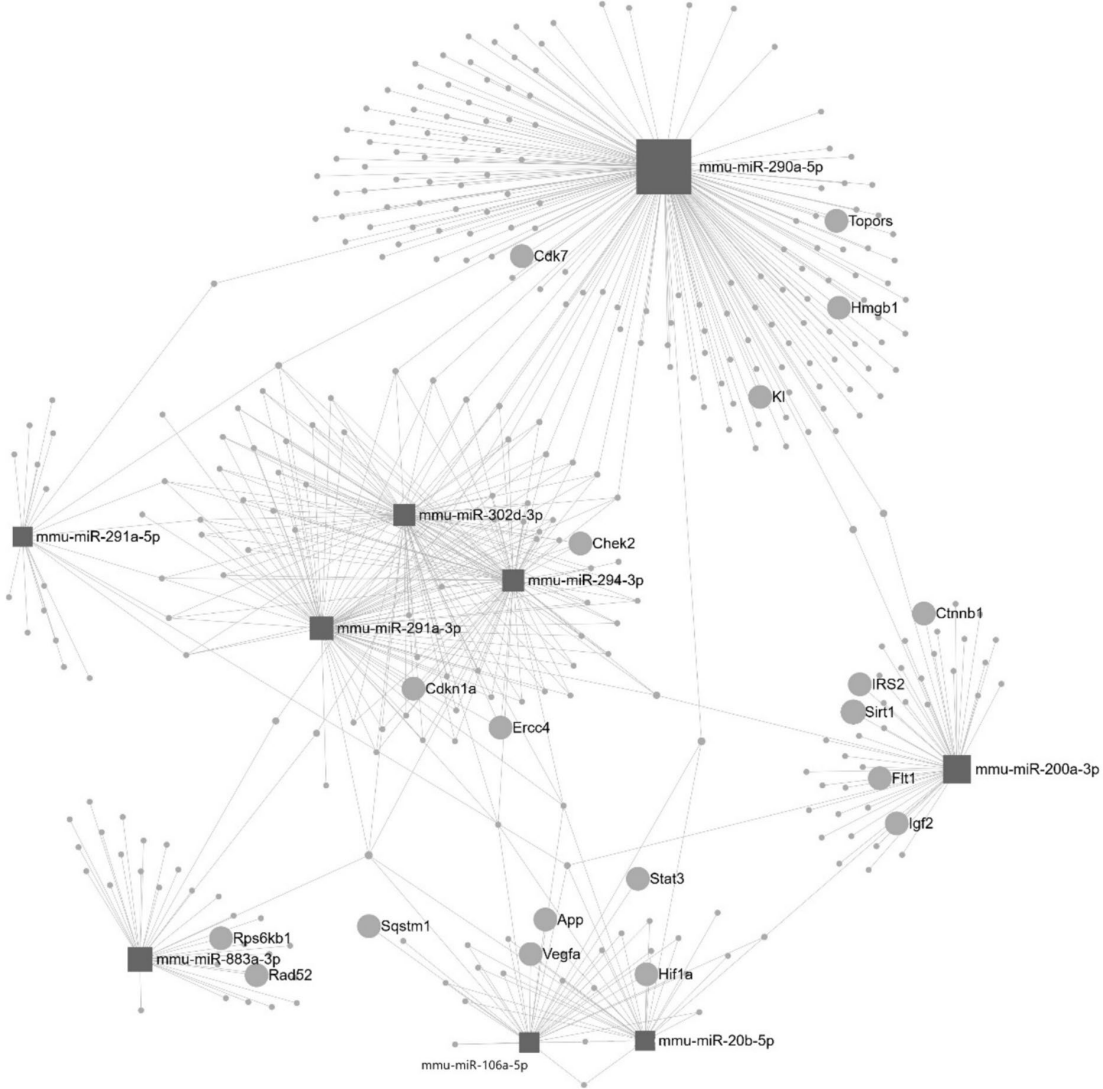
MicroRNA-regulated PPI network (see the text for explanations)

Considering the multiplicity of MSC secretome components, it would be reasonable to suggest multiple targets and pathways of MSC effects. Among various types of EV cargos, microRNAs (miRs) are of particular interest. In longevity-related studies, the miR content of EVs was explored by Yu et al. (2023) and Chen et al. (2024). Yu et al. (2023) evaluated the miRNA landscape of EVs derived from ESCs compared to EVs derived from embryonic fibroblasts. Based on their results, we noticed that over 70% of miRs, which are over-presented in ESC-derived EVs, were found to target longevity-associated genes (LAGs; Tacutu et al. 2018; https://genomics.senescence.info/genes/index.html). Furthermore, we conducted the KEGG analysis which showed that LAGs targeted by the top 20 over-represented miRs, are primarily involved in well-recognized longevity pathways such as FoxO signaling pathway, Insulin resistance, Cellular senescence, PI3K-Akt signaling pathway, Autophagy, Pathways in cancer, Cell cycle, and Apoptosis. Of note, 9 of top 20 over-represented miRs together with their target genes form the continuous miR-regulated protein–protein interaction (PPI) network which includes 19 well-known LAGs (Fig. 2). Moreover, miRs which are differentially represented in ESC-derived EVs, are involved in regulation of all conditions recognized as hallmarks of aging (López-Otín et al. 2013, 2023; Harries 2014). In particular, numerous studies showed the inhibitory effect of EVs on cellular senescence (reviewed by Rudnitsky et al. 2024). In addition to targeting the LAGs, the aforementioned miRs could promote longevity by still unknown mechanisms. For example, miR-708 which, according to the miRPath database (Kehl et al. 2020; https://mpd.bioinf.uni-sb.de/overview.html), does not yet have experimentally validated targets, has been shown to be associated with a longer lifespan in mice (Lee et al. 2017). Consistent with the effects of EVs, administration of murine stem cells extended lifespan in mice, whereas primary embryonic fibroblasts did not display such an effect (Lavasani et al. 2012; Table 1).

In another work, the miR content of EVs extracted from blood plasma of young and old animals was compared (Chen et al. 2024). The authors identified three miRs that were over-represented in EVs from the plasma of young animals: miR-144-3p, miR-149-5p, and miR-455–3p. Our analysis showed that all of them target LAGs (*APP*, *TAU/MAPT*, and *CRTC1*) with anti-longevity action. Accordingly, miR-induced silencing of these genes might have a longevity-promoting effect. It seems plausible that the lifespan-extending effect of EVs from “young” plasma is, in part, attributed to the high levels of aforementioned miRs. It would be attractive to speculate that MSCs from various tissues (Yoshida et al. 2019) were a valuable source of the plasma-derived EVs.

## Concluding remarks and perspectives

MSCs and MSC-derived EVs hold promise for promoting longevity. Notably, MSCs have relatively low immunogenicity and tumorigenicity and their efficacy is only slightly influenced by donor age in case of humans (in contrast to rodents). Yet, while evaluating the effects of MSC transplantation, the possibility of MSCs to undergo cellular senescence should be taken into consideration (Alessio et al. 2023; Siraj et al. 2023). Along with MSCs, other types of stem cells were reported to display health- and lifespan-extending effects in rodents (Lavasani et al. 2012; Yu et al. 2023). It is also true with regard to EVs derived from embryonic stem cells (ESCs) or extracted from blood plasma (Yoshida et al. 2019; Yu et al. 2023). The application of EVs appears to be even more beneficial than stem cell therapy. Of note, the safeness of MSC/EV transplantation in humans was shown in numerous clinical trials, including ARDs (Strauer et al. 2010; Rodríguez-Fuentes et al. 2021; Koda et al. 2024). However, to date, the longevity-promoting effects of MSC/EV therapy have been limited to rodent studies. Direct extrapolation of rodent data to humans is thus far mostly speculative. Indeed, the aging phenotype could significantly differ across the species, mammals included (Rattan 2024). Therefore, the evaluation of MSC/EV therapeutic potential warrants further thorough investigation and is an important point for future longitudinal studies in humans.

Pluripotent Muse cells could be particularly relevant for promoting healthspan (Dezawa 2018). Yet, the age-related aspects of Muse cell biology have not been fully addressed. An important point for future investigations would be the evaluation of the rejuvenation potential of Muse cells, with the application of aging clocks for biological age determination (Muradian and Fraifeld 2024a, b).

## Data Availability

No datasets were generated or analysed during the current study.

## Abbreviations

ARDs: Age-related diseases
ESC: Embryonic stem cell
EVs: Extracellular vesicles
i.p.: Intraperitoneally
i.v.: Intravenously
LAGs: Longevity-associated genes
MiRs: MicroRNAs
MSCs: Mesenchymal stem cells
